# Determining minimal important differences for patient-reported outcome measures in shoulder, lateral elbow, patellar and Achilles tendinopathies using distribution-based methods

**DOI:** 10.1186/s12891-023-06261-9

**Published:** 2023-03-02

**Authors:** Dimitris Challoumas, Andrea Zouvani, Kevin Creavin, Elspeth Murray, Gearoid Crosbie, Nigel Ng, Neal L. Millar

**Affiliations:** School of Infection and Immunity, College of Medicine, Veterinary and Life Sciences, University of Glasgow, Glasgow, UK

**Keywords:** Tendinopathy, Management, Outcome measure, MCID

## Abstract

**Background:**

Minimal important difference (MID) is a concept used inconsistently and arbitrarily in tendinopathy research. Our aim was to determine the MIDs for the most commonly used tendinopathy outcome measures using data-driven approaches.

**Methods:**

Recently published systematic reviews of randomised controlled trials (RCTs) on tendinopathy management were identified and used for extraction of eligible studies through a literature search. Each eligible RCT was used to obtain information on MID where this was used and it also contributed data for the calculation of the baseline pooled standard deviation (SD) for each tendinopathy (shoulder, lateral elbow, patellar and Achilles). The rule of “half SD” was used for the computation of MIDs for patient-reported pain (visual analogue scale, VAS 0–10, single-item questionnaire) and function (multi-item questionnaires) and the rule of “one standard error of measurement (SEM)” was additionally used for the multi-item functional outcome measures.

**Results:**

A total of 119 RCTs were included for the 4 tendinopathies. MID was defined and used by 58 studies (49%) and there were significant inconsistencies amongst studies where the same outcome measure was used as MID. From our data-driven methods the following suggested MIDs were obtained: a) Shoulder tendinopathy, pain VAS (combined) 1.3 points, Constant-Murley score 6.9 (half SD) and 7.0 (one SEM) points; b) lateral elbow tendinopathy, pain VAS (combined) 1.0 point, Disabilities of Arm, Shoulder and Hand questionnaire 8.9 (half SD) and 4.1 (one SEM) points; c) Patellar tendinopathy, pain VAS (combined) 1.2 points, Victorian Institute of Sport Assessment – Patella (VISA-P) 7.3 (half SD) and 6.6 points (one SEM); d) Achilles tendinopathy, pain VAS (combined) 1.1 points, VISA-Achilles (VISA-A) 8.2 (half SD) and 7.8 points (one SEM). The rules of half SD and one SEM produced very similar MIDs except for DASH due to its very high internal consistency. MIDs were also calculated for different pain settings for each tendinopathy.

**Conclusions:**

Our computed MIDs can be used in tendinopathy research to increase consistency. Clearly defined MIDs should be used with consistency in tendinopathy management studies in the future.

**Supplementary Information:**

The online version contains supplementary material available at 10.1186/s12891-023-06261-9.

## Keypoints

We provide proposed MIDs for patient-reported instruments in tendinopathy using data-driven approaches, which had not previously been used for this purpose.

We call for the complicated, yet important concept of MID to receive greater attention in the future from researchers, publishing journals and outcome measure creators.

Both anchor- and data-driven methods have their strengths and drawbacks and a “hybrid” approach using aspects from both may be best.

## Introduction

The concept of minimal clinically important difference (MCID) was introduced by Jaeschke et al. (1989) and was defined as “the smallest difference in score in the domain of interest which patients perceive as beneficial and which would mandate, in the absence of troublesome side effects and excessive cost, a change in the patient's management” [1]. The authors used global ratings of change (GROC) to establish plausible ranges within which the MCID should fall, and their argument for the relevance of MCID was that statistically significant changes during use of instruments assessing interventions do not always translate to clinically important differences. With the constant emergence of patient-reported outcome measures over the last decades, the use of MCID is becoming increasingly relevant and important; however, the concept of MCID in research remains controversial because of its inherent complexity and difficulty defining.

A minimal important difference (MID), which represents the minimum meaningful change of an outcome measure, should not be confused with the smallest/minimum detectable change (SDC or MDC), which is a measure of the measurement error of the instrument [2]. A change in any given score can only be considered to represent a real change when it’s larger than the SDC [2]. A MID should only be referred to as MCID when clinical methods are used for its computation [3]. The term MID will be used throughout the present article as only non-clinical data-driven methods were employed.

In tendinopathy, the existence of a myriad outcome measures and the continuous introduction of new interventions necessitate objective and consistent assessment of their effectiveness. The use of MID is relevant both in research and clinical practice in the following contexts: a) as part of the power calculation in clinical trials; b) as part of strength of evidence assessment in systematic reviews (e.g. assessment of “imprecision” using the GRADE (Grading of Recommendations, Assessment, Development and Evaluations) tool); c) deciding on the effectiveness of assessed interventions in clinical trials, combined with statistical significance; and d) in clinical practice to assess improvement and guide management [4]. In the existing tendinopathy literature, MIDs have mostly been used arbitrarily and inconsistently [5–7].

Various different methods have been used in the literature for determining MIDs. These are largely divided into “within patients” and “across groups of patients” methods or into data-driven (distribution-based) and anchor-based methods. Anchor-based approaches consider patient perspectives (or less commonly clinician perspectives) and they are the most frequently used methods for the computation of MIDs [8]. Data-driven approaches consider changes within individuals, as opposed to changes across groups, and the main one is “the rule of one standard error of measurement (SEM)’’ as proposed by Wyrwich et al. (1999), who validated this method against Jaeschke’s approach and cutpoints [9]. The main advantage of this approach is that it is standardised and the least subjective as it considers the internal consistency of the instrument itself and the baseline standard deviation (SD) of the population of interest and is the least dependent on population characteristics. This was subsequently simplified as “the rule of half SD”, according to which the MID of most instruments or scales is very close to a value of half the baseline SD of the studied population [10–12]. Its main disadvantage compared to anchor-based methods, which usually use GROC as the “anchor”, is that they do not consider the importance of the observed change [13].

Our aim with the present study was two-fold: a) to investigate and present what previously published tendinopathy trials defined as MID and see whether their use has been consistent; and b) to determine MID values for patient-reported pain and commonly used patient-reported function scales for shoulder, lateral elbow, patellar and Achilles tendinopathies separately using data-driven approaches. We expect this will be helpful for future research and clinical practice in tendinopathy, will provide further insights into computation of MID and will enhance consistency and comparability among clinical trials.

## Methods

### Eligibility

Studies were eligible if they were randomised controlled trials (RCTs), included patients with shoulder, lateral elbow, patellar or mid-portion Achilles tendinopathy and reported variability statistics (SD; standard error of mean, SEMean; confidence interval, CI; interquartile range, IQR) of the outcomes of interest at baseline. The tendinopathy locations were chosen as the four most prevalent. Studies on insertional Achilles tendinopathy were excluded as it represents a separate pathological condition.

### Study identification

Eligible studies were identified using previously published systematic reviews of RCTs for each tendinopathy. Searches were conducted separately for each tendinopathy in Medline with the following boolean operators used in “all fields”: a) (Patellar tendin* OR Patellar tendin jumper’s knee) AND (review OR systematic review OR meta-analysis); b) Achilles tendin* OR Achilles tendon*) AND (review OR systematic review OR meta-analysis); c) (Elbow tendin* OR elbow tendon* OR tennis elbow OR lateral epicondyl*) AND (review OR systematic review OR meta-analysis); d) (shoulder tendin* OR shoulder tendon* OR rotator cuff tendin* OR rotator cuff tendon* OR subacromial impingement OR subacromial pain) AND (review OR systematic review OR meta-analysis).

The searches were not systematic as the aim was not to identify all eligible RCTs; we aimed to identify a sufficient number of studies for a sufficiently large, combined population size (at least 200 patients for each instrument). We chose to include RCTs instead of observational studies for two reasons: a) the power calculations used in RCTs was used for the first part of our study looking at definition of MIDs; b) RCTs are generally superior methodologically and their baseline measurements are therefore more likely to be accurate. The largest, recently published systematic reviews of RCTs were selected for each one of the 4 tendinopathies.

### Outcome measures

The primary outcome for which MIDs were to be determined was patient-reported pain (visual analogue scale, VAS, or equivalent). The secondary outcome was function/functional disabilities; for each tendinopathy, the instruments used were those most commonly encountered in the included RCTs.

### Data handling

For each RCT, the following data were extracted and tabulated into Microsoft Excel: a) sample size of each treatment group, b) baseline mean and standard deviation for each outcome measure of interest, and c) what the study authors defined as MID for their power calculation where that was performed. Follow up study data were ignored as they were irrelevant for the purposes of our study.

The MIDs of each outcome measure of interest were calculated as follows:

- for all outcome measures (single-item and multi-item patient-reported) the MID was assumed to be equal to half the pooled SD at baseline based on the rule of half SD [5]; where the reliability of the instrument is very high (which is generally the case for single-item outcome measures, such as pain VAS), using the one SEM rule would result in a very small value of MID and therefore the half SD threshold is a more stringent criterion [10].
- for multi-item patient-reported outcome measures (e.g. Victorian Institute of Sport Assessment, VISA), the one SEM rule was also used to calculate the MID to see how that compares to the corresponding value deriving from the rule of half SD [10]. Cronbach’s alpha was used as an internal consistency measure in the equation.

The SEM is a statistic used to estimate how reliably an instrument measures an individual’s “true score”. The Cronbach’s alpha, which measures the internal consistency of an instrument, is the degree of the inter-relatedness among the items of the instrument [14]. In contrast to test–retest statistics (e.g. intraclass correlation coefficient, ICC), using Cronbach’s alpha is especially useful when the assessed parameters can change over short periods of time [15].

For each tendinopathy separately, pooled SDs of VAS at the most commonly reported specific settings (e.g. at rest, with activity etc.) were also computed and presented separately where there was a sufficiently large pooled population size (n > 200 patients). We standardised pain VAS scores by using an 11-point scale (0–10) as this was the most commonly used scale; where a 0–100 scale was used by studies, reported values were converted to their equivalent on a 0–10 scale by dividing them by “10”.

### Statistical analysis

All variability statistics were converted to SDs. When IQRs or data ranges were reported, the SD was calculated as IQR divided by 1.35 and length of range divided by 4 respectively. When CIs of means were reported, SDs were calculated by dividing the length of the CI by 3.92 and then multiplying by the square root of the sample size. When SEmean was given, this were converted to SD by multiplying it by the square root of the sample size.

Pooled SDs were calculated with the following formula: SD_pooled_ = √(SD_1_^2^[*n*_1_-1]) + (SD_2_^2^[*n*_2_-1]) + … + (SD_*k*_^2^[*n*_*k*_-1]) / (*n*_1_ + *n*_2_ + … + *n*_*k*_ – *k*), where *n* indicates sample size and *k*, the number of samples.

SEM for each multi-item patient-reported outcome measure was calculated with the formula:

SEM = SD√ (1-r_xx_), where r_xx_ is the internal consistency of the scale (Cronbach’s alpha coefficient) and SD the pooled SD for that instrument from the included RCTs. The Cronbach’s alpha coefficient for each instrument was obtained from reliability data in the published literature and where more than one studies reported internal consistency values (e.g. different language versions of the instrument), the average of these values was calculated and used [16–24].

## Results

A total of 119 RCTs (Supplementary Table 1) were included and reviewed for all 4 tendinopathies [25–28]. MID was defined and used by 58 studies (49%), of which only 20% provided a reference to justify their chosen MID (studies that previously used it or studies that computed it with anchor-based methods). Data for each tendinopathy are presented below.

### Shoulder tendinopathy

A total of 33 studies were reviewed, of which 8 (24%) defined and used MIDs [25]. One study used MID for two outcomes. Thirty (30) studies contributed data to pain VAS (106 samples, pooled population 3404 patients). The most common settings where pain was reported were “at rest” (10 studies, 22 samples, pooled population *n* = 567 patients) and “with activity/movement” (13 studies, 28 samples, population *n* = 695 patients). The most commonly used functional instrument was the Constant Murley Score (CMS; 15 studies, 32 samples, pooled population *n* = 1412 patients),

Criteria used for MID were as follows: *n* = 2 pain VAS 1.4 points, *n* = 1 pain VAS 1.3 points, *n* = 1 quick Disabilities of Arm, Shoulder and Hand (qDASH) questionnaire 8 points, *n* = 1 Adolfsson-Lysholm (AL) shoulder assessment score 15 points, *n* = 1 shoulder disability questionnaire (SDQ) 3 points, *n* = 1 shoulder pain and disability index (SPADI) 19.6 points, *n* = 1 CMS 9–10 points, *n* = 1 University of California Los Angeles (UCLA) shoulder score 5 points.

### Lateral elbow tendinopathy

A total of 21 studies were reviewed [26]. Of these, 8 (38%) defined and used MIDs. Twelve (12) studies contributed data to pain VAS (38 samples, pooled population *n* = 1704 patients*)*. The most common settings where pain was reported were “worst” (6 studies, 17 samples, pooled population *n* = 614 patients) and “at rest” (4 studies, 11 samples, pooled population *n* = 361 patients)*.* The most commonly used functional instrument was the Disabilities of the Arm, Shoulder and Hand (DASH) scale (4 studies, 9 samples, pooled population *n* = 316 patients).

Criteria used as MID were as follows: *n* = 1 37% improvement in patient-rated tennis elbow evaluation (PRTEE) score, *n* = 1 between-group difference of 6.8 kg in pain-free grip strength, *n* = 1 13 points improvement in PRTEE, *n* = 1 40% improvement in DASH score, *n* = 1 “50% improvement in lateral epicondylalgia cases in the treatment group vs 10% reduction in the control group”, *n* = 1 “50% improvement from baseline”, *n* = 1 “difference in success rate of 25% with the least effective treatment”, *n* = 1 “effect size of 1 on PRTEE questionnaire”.

### Patellar tendinopathy

A total of 37 studies were reviewed, of which 22 (59%) defined and used MIDs [27]. Three studies used MID for two outcomes. Twenty-seven (27) studies contributed data to pain (92 samples, pooled population *n* = 1736 patients)*.* The most common setting where pain was reported was “with single leg decline squat” (8 studies, 16 samples, pooled population *n* = 306 patients). The most commonly used functional instrument was VISA-Patella (VISA-P; 30 studies, 63 samples, pooled population *n* = 1120 patients).

Criteria used as MID were as follows: *n* = 8 VISA-P 13 points, *n* = 4 VISA-P 15 points, *n* = 4 VISA-P 20 points, *n* = 1 VISA-P 11 points, *n* = 5 pain VAS 2 points, *n* = 1 pain VAS 5 points, *n* = 1 12-item short-form survey (SF-12) 6.8 points, *n* = 1 short-form McGill pain questionnaire (SF-MPQ) 4.54 points.

### Achilles tendinopathy

A total of 28 studies were reviewed; 20 (71%) of them defined and used MID [28]. Nineteen (19) studies contributed data to pain (45 samples, pooled population *n* = 1090 patients) with the most commonly used settings being “pain at rest” (11 studies, 27 samples, pooled population *n* = 569 patients) and “with activity” (4 studies, 9 samples, pooled population *n* = 295 patients). The most commonly used functional instrument was VISA-Achilles (VISA-A; 18 studies, 41 samples, pooled population *n* = 1008 patients).

Criteria used for MID were as follows: *n* = 7 VISA-A 10 points, *n* = 3 VISA-A 20 points, *n* = 2 VISA-A 16 points, *n* = 1 VISA-A 20 points, *n* = 1 pain VAS 1 point, *n* = 1 pain VAS 1.2 points, *n* = 2 pain VAS 10% change, *n* = 1 pain VAS 1.5 points, *n* = 1 pain VAS 2 points, *n* = 1 pain VAS 2–3 points.

Our computed MIDs for each tendinopathy are presented in Table 1, which also shows the method used for the calculation and other relevant data.

**Table 1:** Suggested minimal important difference (MID) for shoulder, lateral elbow, patellar and Achilles tendinopathy

Tendinopathy	Outcome	Instrument	Population	Number of samples	*SD*_*pooled*_	Cronbach’s alpha	Computed MID	Method used
**Shoulder**	Function	***CMS***	*N* = 3404	106	13.7	0.74	6.9	**½** *SD*_*pooled*_
							7.0	1 SEM
	Pain	***Pain VAS (combined)***	*N* = 1412	32	2.5	-	1.3	**½** *SD*_*pooled*_
		***Pain at rest VAS***	*N* = 567	22	2.4	-	1.2	**½** *SD*_*pooled*_
		***Pain with activity VAS***	*N* = 695	28	1.9	-	1.0	**½** *SD*_*pooled*_
		***Pain at night VAS***	*N* = 906	19	3.2	-	1.6	**½** *SD*_*pooled*_
**Elbow**	Function	***DASH***	*N* = 316	9	16.7	0.94	8.9	**½** *SD*_*pooled*_
							4.1	1 SEM
	Pain	***Pain VAS (combined)***	*N* = 1704	38	2.0	-	1.0	**½** *SD*_*pooled*_
		***Pain at rest VAS***	*N* = 361	11	1.4	-	0.7	**½** *SD*_*pooled*_
		***Pain worst VAS***	*N* = 614	17	1.9	-	1.0	**½** *SD*_*pooled*_
**Patellar**	Function	***VISA-P***	*N* = 1120	63	14.5	0.79	7.3	**½** *SD*_*pooled*_
							6.6	1 SEM
	Pain	***Pain VAS (combined)***	*N* = 1736	92	2.3	-	1.2	**½** *SD*_*pooled*_
		***Pain with single decline squat VAS***	*N* = 306	16	2.3	-	1.2	**½** *SD*_*pooled*_
**Achilles**	Function	***VISA-A***	*N* = 1008	41	16.3	0.77	8.2	**½** *SD*_*pooled*_
							7.8	1 SEM
	Pain	***Pain VAS (combined)***	*N* = 1090	45	2.2	-	1.1	**½** *SD*_*pooled*_
		***Pain with activity VAS***	*N* = 295	9	2.1	-	1.1	**½** *SD*_*pooled*_
		***Pain at rest VAS***	*N* = 569	27	2.5	-	1.3	**½** *SD*_*pooled*_

*CMS* Constant Murley Score, *VAS* Visual Analogue scale, *DASH* The Disabilities of the Arm, Shoulder and Hand questionnaire, Victorian Institute of Sport Assessment, VISA score, VISA-P (Patellar) and VISA-A (Achilles), *SD* Standard deviation, *SEM* Standard error of measurement

## Discussion

To our knowledge, this is the first study using data-driven approaches to determine MIDs for tendinopathies. Our computed MIDs can be used along with those previously calculated from anchor-based approaches to guide further relevant research.

The concept of MID remains controversial and poorly used. In the present study, fewer than half of the included RCTs defined and used a MID for their power calculation. Where the same instruments were used, we identified big discrepancies in the values used, e.g. change of VISA-A of 10 points in a study and 20 points in another. Where clinical significance is considered along statistical significance, this discrepancy will have important consequences on what magnitude of within-group or between-groups difference is considered significant. Additionally, this substantially influences the power calculation; a MID in VISA-A of 10 points will result in a much larger minimum population required to be recruited compared to a MID of 20 points.

Proposed MIDs for commonly used patient-reported function instruments in tendinopathy have been previously published using anchor-based methods. In Achilles tendinopathy, a MID of 6.5 points was previously determined by McCormack et al. (2015) for insertional tendinopathy [29]. For mid-portion Achilles tendinopathy, Lagas et al. (2021) used similar anchor-based methods in 64 patients and calculated a VISA-A MID of 14 points at 12 weeks and 7 points at 24 weeks [30]. A recent review article by Murphy et al. (2018) found that, among 46 included studies (randomised and non-randomised), the most commonly used MID for VISA-A was 10 points (*n* = 6), which is in agreement with our findings (*n* = 7) [31]. Other authors arbitrarily suggested a MID of 12 and 20 for VISA-A [32–34].

For patellar tendinopathy, Hernandez-Sanchez et al. (2014) used a similar approach to McCormack et al. (2015) and Lagas et al. (2021) to determine a MID for VISA-P [29, 30, 35]. They administered a VISA-P questionnaire to 98 athletes with patellar tendinopathy along with a 15-point Likert scale; important change was defined as a change of 3 points or more on the Likert scale. The respective VISA-P absolute change was 13 points, which they defined as MID. This was also equivalent to a relative change of 15.4–27% of the baseline score.

MIDs for patient-reported function instruments have not been defined for shoulder tendinopathy specifically. The CMS MID was defined as 10.4 points by Kukkonen et al. (2013) in their prospective study of patients undergoing rotator cuff surgery; they employed a “patient perspective” approach, correlating CMS scores with a simple question: “is the pain better or worse after the operation compared with the pre-operative state?” [36]. Tashjian et al. (2009) determined a MID for pain VAS in patients treated for rotator cuff disease also using an anchor-based method; they found a difference of 1.4 points in pain VAS being a change that patients perceived as clinically beneficial [37].

The DASH questionnaire was the most commonly encountered instrument assessing function in the included trials on lateral elbow tendinopathy. MIDs of 9 or 10 points have commonly been reported for the DASH questionnaire in the literature, which are very close to our MID computed using the rule of half SD (8.9 points) but significantly higher than the one calculated with the one SEM rule (4.1 points). This latter very small figure is due to the very high internal consistency of the instrument which yields a MID that is unrealistically small to represent a clinically important change for patients; therefore we would recommend the use of our result deriving from the rule of half SD (8.9 points). The DASH website recommends a MID of 15 points [38]. Farzad et al. (2020) suggested a DASH MID of 18 for patients with lateral elbow tendinopathy, having correlated DASH scores with GROC scale results in 64 patients [39]. Using a similar method in a larger population (255 patients) suffering from upper limb musculoskeletal disorders, Franchignoni et al. (2014) computed an MID of 10.83 points using an anchor-based method and a SEM of 4.63 using a data-driven method [40]. For the SEM calculation, they used test–retest reliability instead of Cronbach’s alpha coefficient. Finally, Rysstad et al. (2017) found a difference of 4.4 points in the Norwegian version of the DASH questionnaire corresponding to minimal important change, which is similar to our result deriving from the one SEM method [22]. The results of these two last studies are very similar to our own computed MID for the DASH questionnaire deriving from the one SEM method.

Studies have previously attempted to determine MIDs for patient-reported pain in the literature. In a population of 825 patients with chronic musculoskeletal pain (mostly osteoarthritis), Salaffi et al. (2004) reported a change of 1 point in the numerical rating scale (NRS, same as VAS) or a 15% improvement from baseline reflecting a slight clinical improvement based on the patients’ corresponding results in a global impression of change questionnaire and they defined this as MID [41]. However, the authors found that a change of 2 points in the pain NRS or a 33% improvement from baseline was equivalent to a “much better” outcome in the global impression of change questionnaire. Farrar et al. (2001) used a data-driven approach and found that, having used data from 2724 patients with diabetic neuropathy from 10 RCTs, a reduction of 2 points (or 30%) in pain VAS was equivalent to a “much improved” and “very much improved” outcome on GROC scales [42]. Although the authors refer to their approach as data-driven, we argue that this is a hybrid approach as they use pooled data from several studies (data-driven approach) as well as GROC scale results (anchor-based approach). We would encourage the use of different MIDs for each pain setting (e.g. with activity, with sports, at rest etc.) as we argue these represent different outcome measures.

Our data-driven methods do not come without limitations. In contrast to the more commonly employed anchor-based approaches that correlate instrument scores with patient satisfaction/improvement questionnaires, it is based purely on variability statistics of the population of interest and the intrinsic characteristics of the instrument. Indeed, VISA-A and VISA-P for example have low-quality evidence for their internal consistency. However, it eliminates subjectivity that comes with simple patient satisfaction/improvement questionnaires and, additionally, we have used large pooled populations of the same condition for each instrument to make the SD as representative of the population of interest as possible. The validity of previously used methods that utilise GROC is questionable as this may not be the best external standard of change; additionally, studies using such patient perspective methods usually have a small population which may not be representative of the wider population of interest, and finally, there may be recall bias or patients not understanding the context of improvement [29, 40]. To complicate things further, other parameters should ideally be considered for the application of MIDs, including, but not limited to, age, nature, chronicity and severity of disease, potential for improvement, type of intervention and follow up time point [40, 43]. Additionally, although we identified a large number of trials from recently published systematic reviews for each tendinopathy, our search was not systematic, therefore not all eligible studies in the literature have been included.

Finally, even though in reality MIDs may be different at various follow up time points for the same population undergoing the same intervention, setting specific MIDs for each time point will only overcomplicate things. Ranges of MIDs instead of specific values have also been suggested, which would also lead to confusion and inconsistent use, especially when they are wide [40]. Our computed MIDs can be used by clinicians and researchers in the future as they derive from reliable methodology. Going forward, triangulation methods using both anchor-based and distribution-based approaches that will produce either single values or small ranges would be the most accurate determination of MIDs. This should be further re-inforced with Delphi studies including expert clinicians involved in the management of tendinopathy and tendinopathy patient groups [3].

## Conclusions

We demonstrated that MIDs are being used inconcistently in tendinopathy research. We used data-driven approaches and computed MIDs for commonly used outcome measures in shoulder, lateral elbow, patellar and Achilles tendinopathies and these can be used in both clinical and research settings. In the future, more attention should be given to the use of MIDs and their computation through the use of both anchor-based and data-driven approaches.

## Supplementary Information

**Additional file 1.**

## Data Availability

Data is available from DC upon request.
