# Supplementary material for: Determining minimal important differences for patient-reported outcome measures in shoulder, lateral elbow, patellar and Achilles tendinopathies using distribution-based methods
Source: BMC Musculoskelet Disord. 2023 Mar 2;24:158. doi: 10.1186/s12891-023-06261-9 (PMC9979571; doi:10.1186/s12891-023-06261-9)
Supplement: Supplementary file 1 — Additional file 1. [file 12891_2023_6261_MOESM1_ESM.docx]

**Included studies**

***Shoulder tendinopathy RCTs from Dong et al. (2015)***

1. Aktas I, Akgun K, Cakmak B. Therapeutic effect of pulsed electromagnetic field in conservative treatment of subacromial impingement syndrome. Clin Rheumatol 2007; 26:1234–1239.
2. Galace De Freitas D, Marcondes FB, Monteiro RL, et al. Pulsed electromagnetic field and exercises in patients with shoulder impingement syndrome: a randomized, double-blind, placebo-controlled clinical trial. Arch Phys Med Rehabil 2014; 95:345–352.
3. Senbursa G, Baltaci G, Atay A. Comparison of conservative treatment with and without manual physical therapy for patients with shoulder impingement syndrome: a prospective, randomized clinical trial. Knee Surg Sports Traumatol Arthrosc 2007; 15:915–921.
4. Bang MD, Deyle GD. Comparison of supervised exercise with and without manual physical therapy for patients with shoulder impingement syndrome. J Orthop Sports Phys Ther 2000; 30:126–137.
5. Conroy DE, Hayes KW. The effect of joint mobilization as a component of comprehensive treatment for primary shoulder impingement syndrome. J Orthop Sports Phys Ther 1998; 28:3–14.
6. Cook C, Learman K, Houghton S, et al. The addition of cervical unilateral posterior-anterior mobilisation in the treatment of patients with shoulder impingement syndrome: a randomised clinical trial. Man Ther 2014; 19:18–24.
7. Johansson K, Bergstrom A, Schroder K, et al. Subacromial corticosteroid injection or acupuncture with home exercises when treating patients with subacromial impingement in primary care-a randomized clinical trial. Fam Pract 2011; 28:355–365.
8. Hong JY, Yoon SH, Moon DJ, et al. Comparison of high- and low-dose corticosteroid in subacromial injection for periarticular shoulder disorder: A randomized, triple-blind, placebo-controlled trial. Arch Phys Med Rehabil 2011; 92:1951–1960.
9. Min KS, Pierre St, Ryan P, et al. A double-blind randomized controlled trial comparing the effects of subacromial injection with corticosteroid versus NSAID in patients with shoulder impingement syndrome. J Shoulder Elbow Surg 2013; 22:595–601.
10. Penning LIF, De Bie RA, Walenkamp GHIM. The effectiveness of injections of hyaluronic acid or corticosteroid in patients with subacromial impingement: a three-arm randomised controlled trial. J Bone Joint Surg Br 2012; 94:1246–1252.
11. Kim YS, Park JY, Lee CS, et al. Does hyaluronate injection work in shoulder disease in early stage? A multicenter, randomized, single blind and open comparative clinical study. J Shoulder Elbow Surg 2012; 21:722–727.
12. Karthikeyan S, Kwong HT, Upadhyay PK, et al. A double-blind randomised controlled study comparing subacromial injection of tenoxicam or methylprednisolone in patients with subacromial impingement. J Bone Joint Surg Br 2010; 92:77–82.
13. Celik D, Atalar AC, Guclu A, et al. The contribution of subacromial injection to the conservative treatment of impingement syndrome. Acta Orthop Traumatol Turc 2009; 43:331–335.
14. Akgun K, Birtane M, Akarirmak U. Is local subacromial corticosteroid injection beneficial in subacromial impingement syndrome? Clin Rheumatol 2004; 23:496–500.
15. Barra Lopez ME, Lopez de Celis C, Fernandez Jentsch G, et al. Effectiveness of diacutaneous fibrolysis for the treatment of subacromial impingement syndrome: a randomised controlled trial. Man Ther 2013; 18:418–424.
16. Holmgren T, Hallgren HB, Oberg B, et al. Effect of specific exercise strategy on need for surgery in patients with subacromial impingement syndrome: randomised controlled study. BMJ (Online) 2012; 344:
17. Beaudreuil J, Lasbleiz S, Richette P, et al. Assessment of dynamic humeral centering in shoulder pain with impingement syndrome: a randomised clinical trial. Ann Rheum Dis 2011; 70:1613–1618.
18. Baskurt Z, Baskurt F, Gelecek N, et al. The effectiveness of scapular stabilization exercise in the patients with subacromial impingement syndrome. J Back Musculoskelet Rehabil 2011; 24:173–179.
19. Akyol Y, Ulus Y, Durmus D, et al. Effectiveness of microwave diathermy on pain, functional capacity, muscle strength, quality of life, and depression in patients with subacromial impingement syndrome: a randomized placebo-controlled clinical study. Rheumatol Int 2012; 32:3007–3016.
20. Calis HT, Berberoglu N, Calis M. Are ultrasound, laser and exercise superior to each other in the treatment of subacromial impingement syndrome? A randomized clinical trial. Eur J Phys Rehabil Med 2011; 47:375–380.
21. Abrisham SMJ, Kermani-Alghoraishi M, Ghahramani R, et al. Additive effects of low-level laser therapy with exercise on subacromial syndrome: a randomised, double-blind, controlled trial. Clin Rheumatol 2011; 30:1341–1346.
22. Dogan SK, Saime A, Evcik D. The effectiveness of low laser therapy in subacromial impingement syndrome: a randomized placebo controlled double-blind prospective study. Clinics 2010; 65:1019–1022.
23. Yeldan I, Cetin E, Razak Ozdincler A. The effectiveness of low-level laser therapy on shoulder function in subacromial impingement syndrome. Disabil Rehabil 2009; 31:935–940.
24. Bal A, Eksioglu E, Gurcay E, et al. Low-level laser therapy in subacromial impingement syndrome. Photomed Laser Surg 2009; 27:31–36.
25. Engebretsen K, Grotle M, Bautz-Holter E, et al. Radial extracorporeal shockwave treatment compared with supervised exercises in patients with subacromial pain syndrome: single blind randomised study. BMJ 2009; 339:b3360.
26. Vas J, Ortega C, Olmo V, et al. Single-point acupuncture and physiotherapy for the treatment of painful shoulder: a multicentre randomized controlled trial. Rheumatology 2008; 47:887–893.
27. Haahr JP, Ostergaard S, Dalsgaard J, et al. Exercises versus arthroscopic decompression in patients with subacromial impingement: a randomised, controlled study in 90 cases with a one year follow up. Ann Rheum Dis 2005; 64:760–764.
28. Husby T, Haugstvedt JR, Brandt M, et al. Open versus arthroscopic subacromial decompression: a prospective, randomized study of 34 patients followed for 8 years. Acta Orthop Scand 2003; 74:408–414.
29. Henkus HE, De Witte PB, Nelissen RGHH, et al. Bursectomy compared with acromioplasty in the management of subacromial impingement syndrome: a prospective randomised study. J Bone Joint Surg Br 2009; 91:504–510.
30. Lu Y, Zhang Q, Zhu Y, et al. Is radiofrequency treatment effective for shoulder impingement syndrome? A prospective randomized controlled study. J Shoulder Elbow Surg 2013; 22:1488–1494.
31. Everts PA, Devilee RJJ, Brown Mahoney C, et al. Exogenous application of platelet-leukocyte gel during open subacromial decompression contributes to improved patient outcome: a prospective randomized double-blind study. Eur Surg Res 2008; 40:203–210.
32. Simsek HH, Balki S, Keklik SS, Ozturk H, Elden H. Does Kinesio taping in addition to exercise therapy improve the outcomes in subacromial impingement syndrome? A randomized, double-blind, controlled clinical trial. Acta Orthopaedica et Traumatologica Turcica 2013; 47:104–110.
33. Lombardi I, Jr, Magri AG, Fleury AM, Da Silva AC, Natour J. Progressive resistance training in patients with shoulder impingement syndrome: A randomized controlled trial. Arthritis Care and Research 2008; 59:615–622.

**Lateral elbow tendinopathy RCTs from Karanasios et al. (2021)**

1. Bisset L, Beller E, Jull G, et al. Mobilisation with movement and exercise, corticosteroid

injection, or wait and see for tennis elbow: randomised trial. BMJ 2006;333:939.

1. Cherry E, Agostinucci J, McLinden J. The effect of cryotherapy and exercise on lateral

epicondylitis: a controlled randomised study. Int J Ther Rehabil 2012;19:641–50.

1. Coombes BK, Bisset L, Brooks P, et al. Effect of corticosteroid injection, physiotherapy,

or both on clinical outcomes in patients with unilateral lateral epicondylalgia: a

randomized controlled trial. JAMA 2013;309:461–9.

1. Dale LM, Mikuski C, Miller J. Outcomes of a pilates-based intervention for individuals

with lateral epicondylosis: a pilot study. Work 2015;53:163–74.

1. Drechsler WI, Knarr JF, Snyder-Mackler L. A comparison of two treatment regimens for lateral epicondylitis: a randomized trial of clinical interventions. J Sport Rehabil

1997;6:226–34.

1. Luginbühl R, Brunner F, Schneeberger AG. No effect of forearm band and extensor

strengthening exercises for the treatment of tennis elbow: a prospective randomised

study. Chir Organi Mov 2008;91:35–40.

1. Martinez-Silvestrini JA, Newcomer KL, Gay RE, et al. Chronic lateral epicondylitis:

comparative effectiveness of a home exercise program including stretching alone versus stretching supplemented with eccentric or concentric strengthening. J Hand Ther 2005;18:411–34.

1. Murtezani A, Ibraimi Z, Vllasolli TO, et al. Exercise and therapeutic ultrasound compared with corticosteroid injection for chronic lateral epicondylitis: a randomized controlled trial. Ortop Traumatol Rehabil 2015;17:351–7.
2. Nagrale AV, Herd CR, Ganvir S, et al. Cyriax physiotherapy versus phonophoresis with supervised exercise in subjects with lateral epicondylalgia: a randomized clinical trial. J Man Manip Ther 2009;17:171–8.
3. Olaussen M, Holmedal Øystein, Mdala I, et al. Corticosteroid or placebo injection combined with deep transverse friction massage, Mills manipulation, stretching and eccentric exercise for acute lateral epicondylitis: a randomised, controlled trial. BMC Musculoskelet Disord 2015;16:122.
4. Park J-Y, Park H-K, Choi J-H, et al. Prospective evaluation of the effectiveness of a home-based program of isometric strengthening exercises: 12-month follow-up. Clin Orthop Surg 2010;2:173–8.
5. Peterson M, Butler S, Eriksson M, et al. A randomized controlled trial of exercise versus wait-list in chronic tennis elbow (lateral epicondylosis). Ups J Med Sci 2011;116:269–79.
6. Peterson M, Butler S, Eriksson M, et al. A randomized controlled trial of eccentric vs. concentric graded exercise in chronic tennis elbow (lateral elbow tendinopathy). Clin Rehabil 2014;28:862–72.
7. Pienimäki T, Karinen P, Kemilä T, et al. Long-term follow-up of conservatively treated chronic tennis elbow patients. A prospective and retrospective analysis. Scand J Rehabil Med 1998;30:159–66.
8. Pienimäki TT, Tarvainen TK, Siira PT, et al. Progressive strengthening and stretching exercises and ultrasound for chronic lateral epicondylitis. Physiotherapy 1996;82:522–30.
9. Sethi K, Noohu MM. Scapular muscles strengthening on pain, functional outcome and muscle activity in chronic lateral epicondylalgia. J Orthop Sci 2018;23:777–82.
10. Smidt N, van der Windt DAWM, Assendelft WJJ, et al. Corticosteroid injections,

physiotherapy, or a wait-and-see policy for lateral epicondylitis: a randomised

controlled trial. Lancet 2002;359:657–62.

1. Söderberg J, Grooten WJ, Äng BO. Effects of eccentric training on hand strength in

subjects with lateral epicondylalgia: a randomized-controlled trial. Scand J Med Sci Sports 2012;22:797–803.

1. Stasinopoulos D, Stasinopoulos I. Comparison of effects of eccentric training,

eccentric-concentric training, and eccentric-concentric training combined with isometric contraction in the treatment of lateral elbow tendinopathy. J Hand Ther 2017;30:13–19.

1. Struijs PAA, Damen P-J, Bakker EWP, et al. Manipulation of the wrist for management of lateral epicondylitis: a randomized pilot study. Phys Ther 2003;83:608–16.
2. Struijs PAA, Kerkhoffs GMMJ, Assendelft WJJ, et al. Conservative treatment of lateral epicondylitis: brace versus physical therapy or a combination of both-a randomized clinical trial. Am J Sports Med 2004;32:462–9.
3. Svernlöv B, Adolfsson L. Non-operative treatment regime including eccentric training for lateral humeral epicondylalgia. Scand J Med Sci Sports 2001;11:328–34.
4. Tonks JH. Evaluation of short-term conservative treatment in patients with tennis elbow (lateral epicondylitis) : a prospective randomised, assessor-blinded trial. University of Central Lancashire, 2012.
5. Tonks JH, Pai SK, Murali SR. Steroid injection therapy is the best conservative treatment for lateral epicondylitis: a prospective randomised controlled trial. Int J Clin Pract 2007;61:240–6.
6. Tyler TF, Thomas GC, Nicholas SJ, et al. Addition of isolated wrist extensor eccentric exercise to standard treatment for chronic lateral epicondylosis: a prospective randomized trial. J Shoulder Elbow Surg 2010;19:917–22.
7. Viswas R, Ramachandran R, Korde Anantkumar P. Comparison of effectiveness of supervised exercise program and Cyriax physiotherapy in patients with tennis elbow (lateral epicondylitis): a randomized clinical trial. ScientificWorldJournal 2012;2012:1–8.
8. Vuvan V, Vicenzino B, Mellor R, et al. Unsupervised isometric exercise versus Wait-and- See for lateral elbow tendinopathy. Med Sci Sports Exerc 2020;52:287–95.
9. Wen DY, Schultz BJ, Schaal B, et al. Eccentric strengthening for chronic lateral epicondylosis: a prospective randomized study. Sports Health 2011;3:500.
10. Yelland M, Rabago D, Ryan M, et al. Prolotherapy injections and physiotherapy used singly and in combination for lateral epicondylalgia: a single-blinded randomised clinical trial. BMC Musculoskelet Disord 2019;20:509.

***Achilles tendinopathy RCTs from van der Vlist et al. (2020)***

1. Bell KJ, Fulcher ML, Rowlands DS, et al. . Impact of autologous blood injections in treatment of mid-portion achilles tendinopathy: double blind randomised controlled trial. BMJ 2013;346:f2310. 10.1136/bmj.f2310
2. Beyer R, Kongsgaard M, Hougs Kjær B, et al. . Heavy slow resistance versus eccentric training as treatment for achilles tendinopathy: a randomized controlled trial. Am J Sports Med 2015;43:1704–11. 10.1177/0363546515584760
3. Boesen AP, Hansen R, Boesen MI, et al. . Effect of high-volume injection, platelet-rich plasma, and sham treatment in chronic midportion achilles tendinopathy: a randomized double-blinded prospective study. Am J Sports Med 2017;45:2034–43. 10.1177/0363546517702862
4. de Jonge S, de Vos RJ, Van Schie HTM, et al. . One-year follow-up of a randomised controlled trial on added splinting to eccentric exercises in chronic midportion achilles tendinopathy. Br J Sports Med 2010;44:673–7. 10.1136/bjsm.2008.052142
5. de Jonge S, de Vos RJ, Weir A, et al. . One-year follow-up of platelet-rich plasma treatment in chronic achilles tendinopathy: a double-blind randomized placebo-controlled trial. Am J Sports Med 2011;39:1623–9. 10.1177/0363546511404877
6. Herrington L, McCulloch R. The role of eccentric training in the management of achilles tendinopathy: a pilot study. Physical Therapy in Sport 2007;8:191–6. 10.1016/j.ptsp.2007.07.001
7. Hutchison AM, Pallister I, Evans RM, et al. . Intense pulsed light treatment of chronic mid-body achilles tendinopathy: a double blind randomised placebo-controlled trial. Bone Joint J 2013;95-B:504–9. 10.1302/0301-620X.95B4.30558
8. Krogh TP, Ellingsen T, Christensen R, et al. . Ultrasound-Guided injection therapy of achilles tendinopathy with platelet-rich plasma or saline: a randomized, blinded, placebo-controlled trial. Am J Sports Med 2016;44:1990–7. 10.1177/0363546516647958
9. Lynen N, De Vroey T, Spiegel I, et al. . Comparison of peritendinous hyaluronan injections versus extracorporeal shock wave therapy in the treatment of painful achilles' tendinopathy: a randomized clinical efficacy and safety study. Arch Phys Med Rehabil 2017;98:64–71. 10.1016/j.apmr.2016.08.470 [
10. Munteanu SE, Scott LA, Bonanno DR, et al. . Effectiveness of customised foot orthoses for achilles tendinopathy: a randomised controlled trial. Br J Sports Med 2015;49:989–94. 10.1136/bjsports-2014-093845
11. Pearson J, Rowlands D, Highet R. Autologous blood injection to treat achilles tendinopathy? A randomized controlled trial. J Sport Rehabil 2012;21:218–24. 10.1123/jsr.21.3.218
12. Rompe JD, Furia J, Maffulli N. Eccentric loading compared with shock wave treatment for chronic insertional achilles tendinopathy: a randomized, controlled trial. J Bone Joint Surg Am 2008;90:52–61. 10.2106/JBJS.F.01494 [
13. Rompe JD, Furia J, Maffulli N. Eccentric loading versus eccentric loading plus shock-wave treatment for midportion achilles tendinopathy: a randomized controlled trial. Am J Sports Med 2009;37:463–70. 10.1177/0363546508326983
14. Rompe JD, Nafe B, Furia JP, et al. . Eccentric loading, shock-wave treatment, or a wait-and-see policy for tendinopathy of the main body of tendo achillis: a randomized controlled trial. Am J Sports Med 2007;35:374–83. 10.1177/0363546506295940
15. Roos EM, Engström M, Lagerquist A, et al. . Clinical improvement after 6 weeks of eccentric exercise in patients with mid-portion achilles tendinopathy -- a randomized trial with 1-year follow-up. Scand J Med Sci Sports 2004;14:286–95. 10.1111/j.1600-0838.2004.378.x
16. Silbernagel KG, Thomeé R, Eriksson BI, et al. . Continued sports activity, using a pain-monitoring model, during rehabilitation in patients with achilles tendinopathy: a randomized controlled study. Am J Sports Med 2007;35:897–906. 10.1177/0363546506298279
17. Stevens M, Tan C-W. Effectiveness of the alfredson protocol compared with a lower repetition-volume protocol for midportion achilles tendinopathy: a randomized controlled trial. J Orthop Sports Phys Ther 2014;44:59–67. 10.2519/jospt.2014.4720 [PubMed] [CrossRef] [Google Scholar]
18. Tumilty S, Mani R, Baxter GD. Photobiomodulation and eccentric exercise for achilles tendinopathy: a randomized controlled trial. Lasers Med Sci 2016;31:127–35. 10.1007/s10103-015-1840-4
19. Tumilty S, McDonough S, Hurley DA, et al. . Clinical effectiveness of low-level laser therapy as an adjunct to eccentric exercise for the treatment of achilles' tendinopathy: a randomized controlled trial. Arch Phys Med Rehabil 2012;93:733–9. 10.1016/j.apmr.2011.08.049
20. Yelland MJ, Sweeting KR, Lyftogt JA, et al. . Prolotherapy injections and eccentric loading exercises for painful achilles tendinosis: a randomised trial. Br J Sports Med 2011;45:421–8. 10.1136/bjsm.2009.057968
21. Zhang B-meng, Zhong L-wei, Xu S-wei, et al. . Acupuncture for chronic achilles tendnopathy: a randomized controlled study. Chin J Integr Med 2013;19:900–4. 10.1007/s11655-012-1218-4
22. Auclair J, Georges M, Grapton X, et al. . A double-blind controlled multicenter study of percutaneous niflumic acid gel and placebo in the treatment of achilles heel tendinitis. Eur J Rheumatol Inflamm 1991;46:782–8.
23. Njawaya MM, Moses B, Martens D, et al. . Ultrasound guidance does not improve the results of shock wave for plantar fasciitis or calcific achilles tendinopathy: a randomized control trial. Clin J Sport Med 2018;28:21–7. 10.1097/JSM.0000000000000430
24. Usuelli FG, Grassi M, Maccario C, et al. . Intratendinous adipose-derived stromal vascular fraction (SVF) injection provides a safe, efficacious treatment for achilles tendinopathy: results of a randomized controlled clinical trial at a 6-month follow-up. Knee Surg Sports Traumatol Arthrosc 2018;26:2000–10. 10.1007/s00167-017-4479-9
25. Ebbesen BH, Mølgaard CM, Olesen JL, et al. . No beneficial effect of polidocanol treatment in achilles tendinopathy: a randomised controlled trial. Knee Surg Sports Traumatol Arthrosc 2018;26:2038–44. 10.1007/s00167-017-4675-7
26. Heinemeier KM, Øhlenschlæger TF, Mikkelsen UR, et al. . Effects of anti-inflammatory (NSAID) treatment on human tendinopathic tissue. J Appl Physiol 2017;123:1397–405. 10.1152/japplphysiol.00281.2017
27. Morrison RJM, Brock TM, Reed MR, et al. . Radiofrequency microdebridement versus surgical decompression for achilles tendinosis: a randomized controlled trial. J Foot Ankle Surg 2017;56:708–12. 10.1053/j.jfas.2017.01.049
28. Silbernagel KG, Thomeé R, Thomeé P, et al. . Eccentric overload training for patients with chronic achilles tendon pain--a randomised controlled study with reliability testing of the evaluation methods. Scand J Med Sci Sports 2001;11:197–206. 10.1034/j.1600-0838.2001.110402.x

***Patellar tendinopathy RCTs from Challoumas et al. (2021)***

1. Abat F, Sánchez-Sánchez JL, Martín-Nogueras AM, Calvo-Arenillas JI, Yajeya J, Méndez-Sánchez R, Monllau JC, Gelber PE. Randomized controlled trial comparing the effectiveness of the ultrasound-guided galvanic electrolysis technique (USGET) versus conventional electro-physiotherapeutic treatment on patellar tendinopathy. J Exp Orthop. 2016;3(1):34.
2. Agergaard AS, Svensson RB, Malmgaard-Clausen NM, Couppé C, Hjortshoej MH, Doessing S, Kjaer M, Magnusson SP. Clinical Outcomes, Structure, and Function Improve With Both Heavy and Moderate Loads in the Treatment of Patellar Tendinopathy: A Randomized Clinical Trial. Am J Sports Med. 2021 Mar;49(4):982-993.
3. Bahr R, Fossan B, Løken S, Engebretsen L. Surgical treatment compared with eccentric training for patellar tendinopathy (Jumper's Knee). A randomized, controlled trial. J Bone Joint Surg Am. 2006;88(8):1689-98.
4. Breda SJ, Oei EHG, Zwerver J, Visser E, Waarsing E, Krestin GP, de Vos RJ. Effectiveness of progressive tendon-loading exercise therapy in patients with patellar tendinopathy: a randomised clinical trial. Br J Sports Med. 2021;55(9):501-509.
5. Cannell LJ, Taunton JE, Clement DB, Smith C, Khan KM. A randomised clinical trial of the efficacy of drop squats or leg extension/leg curl exercises to treat clinically diagnosed jumper's knee in athletes: pilot study. Br J Sports Med. 2001;35(1):60-4.
6. Clarke AW, Alyas F, Morris T, Robertson CJ, Bell J, Connell DA. Skin-derived tenocyte-like cells for the treatment of patellar tendinopathy. Am J Sports Med. 2011;39(3):614-23.
7. de Vries A, Zwerver J, Diercks R, Tak I, van Berkel S, van Cingel R, van der Worp H, van den Akker-Scheek I. Effect of patellar strap and sports tape on pain in patellar tendinopathy: A randomized controlled trial. Scand J Med Sci Sports. 2016;26(10):1217-24.
8. Dragoo JL, Wasterlain AS, Braun HJ, Nead KT. Platelet-rich plasma as a treatment for patellar tendinopathy: a double-blind, randomized controlled trial. Am J Sports Med. 2014;42(3):610-8.
9. Frohm A, Saartok T, Halvorsen K, Renström P. Eccentric treatment for patellar tendinopathy: a prospective randomised short-term pilot study of two rehabilitation protocols. Br J Sports Med. 2007;41(7):e7.
10. Hoksrud A, Ohberg L, Alfredson H, Bahr R. Ultrasound-guided sclerosis of neovessels in painful chronic patellar tendinopathy: a randomized controlled trial. Am J Sports Med. 2006 Nov;34(11):1738-46.
11. Holden S, Lyng K, Graven-Nielsen T, Riel H, Olesen JL, Larsen LH, Rathleff MS. Isometric exercise and pain in patellar tendinopathy: A randomized crossover trial. J Sci Med Sport. 2020;23(3):208-214.
12. Jonsson P, Alfredson H. Superior results with eccentric compared to concentric quadriceps training in patients with jumper's knee: a prospective randomised study. Br J Sports Med. 2005;39(11):847-50.

# Kaux JF, Bornheim S, Dardenne N, Deroisy R, Samson A, Roberjot M, Croisier JL. Comparison between platelet-rich plasma injections and hyaluronic acid injections in the treatment of patellar tendinopathies: a randomized trial. Muscle Lig Tend J 2019; 9(3): 322-327.

1. Kaux JF, Libertiaux V, Croisier JL, Crielaard JM. Platelet-rich plasma (PRP) to treat chronic patellar tendinopathies: comparison of a single versus two closely-timed injections. Muscles Ligaments Tendons J. 2016;5(4):297-8.
2. Kongsgaard M, Kovanen V, Aagaard P, Doessing S, Hansen P, Laursen AH, Kaldau NC, Kjaer M, Magnusson SP. Corticosteroid injections, eccentric decline squat training and heavy slow resistance training in patellar tendinopathy. Scand J Med Sci Sports. 2009;19(6):790-802.
3. Lee WC, Ng GY, Zhang ZJ, Malliaras P, Masci L, Fu SN. Changes on Tendon Stiffness and Clinical Outcomes in Athletes Are Associated With Patellar Tendinopathy After Eccentric Exercise. Clin J Sport Med. 2017;30(1):25-32.
4. López-Royo MP, Ríos-Díaz J, Galán-Díaz RM, Herrero P, Gómez-Trullén EM. A Comparative Study of Treatment Interventions for Patellar Tendinopathy: A Randomized Controlled Trial. Arch Phys Med Rehabil. 2021;102(5):967-975.
5. Pietrosimone LS, Blackburn JT, Wikstrom EA, Berkoff DJ, Docking SI, Cook J, Padua DA. Landing biomechanics are not immediately altered by a single-dose patellar tendon isometric exercise protocol in male athletes with patellar tendinopathy: A single-blinded randomized cross-over trial. Phys Ther Sport. 2020;46:177-185.
6. Resteghini P, Khanbhai TA, Mughal S, Sivardeen Z. Double-Blind Randomized Controlled Trial: Injection of Autologous Blood in the Treatment of Chronic Patella Tendinopathy-A Pilot Study. Clin J Sport Med. 2016;26(1):17-23.
7. Rigby JH, Mortensen BB, Draper DO. Wireless Versus Wired Iontophoresis for Treating Patellar Tendinopathy: A Randomized Clinical Trial. J Athl Train. 2015;50(11):1165-73.
8. Rio E, Kidgell D, Purdam C, Gaida J, Moseley GL, Pearce AJ, Cook J. Isometric exercise induces analgesia and reduces inhibition in patellar tendinopathy. Br J Sports Med. 2015;49(19):1277-83.
9. Rio E, van Ark M, Docking S, Moseley GL, Kidgell D, Gaida JE, van den Akker-Scheek I, Zwerver J, Cook J. Isometric Contractions Are More Analgesic Than Isotonic Contractions for Patellar Tendon Pain: An In-Season Randomized Clinical Trial. Clin J Sport Med. 2017;27(3):253-259.
10. Rodas G, Soler-Rich R, Rius-Tarruella J, Alomar X, Balius R, Orozco L, Masci L, Maffulli N. Effect of Autologous Expanded Bone Marrow Mesenchymal Stem Cells or Leukocyte-Poor Platelet-Rich Plasma in Chronic Patellar Tendinopathy (With Gap >3 mm): Preliminary Outcomes After 6 Months of a Double-Blind, Randomized, Prospective Study. Am J Sports Med. 2021;49(6):1492-1504.
11. Scott A, LaPrade RF, Harmon KG, Filardo G, Kon E, Della Villa S, Bahr R, Moksnes H, Torgalsen T, Lee J, Dragoo JL, Engebretsen L. Platelet-Rich Plasma for Patellar Tendinopathy: A Randomized Controlled Trial of Leukocyte-Rich PRP or Leukocyte-Poor PRP Versus Saline. Am J Sports Med. 2019;47(7):1654-1661.
12. Stasinopoulos D, Stasinopoulos I. Comparison of effects of exercise programme, pulsed ultrasound and transverse friction in the treatment of chronic patellar tendinopathy. Clin Rehabil. 2004;18(4):347-52.
13. Steunebrink M, Zwerver J, Brandsema R, Groenenboom P, van den Akker-Scheek I, Weir A. Topical glyceryl trinitrate treatment of chronic patellar tendinopathy: a randomised, double-blind, placebo-controlled clinical trial. Br J Sports Med. 2013;47(1):34-9.
14. Taunton J, Taunton KM, Karim KM. Treatment of patellar tendinopathy with extracorporeal shock wave therapy. BC Med J 2003; 45(10).
15. Thijs KM, Zwerver J, Backx FJG, et al. Effectiveness of shockwave treatment combined with eccentric training for patellar tendinopathy: a double-blinded randomised study. Clin J Sport Med. 2017;27:89-96.
16. van Ark M, Cook JL, Docking SI, Zwerver J, Gaida JE, van den Akker-Scheek I, Rio E. Do isometric and isotonic exercise programs reduce pain in athletes with patellar tendinopathy in-season? A randomised clinical trial. J Sci Med Sport. 2016;19(9):702-6.
17. van der Worp H, Zwerver J, Hamstra M, van den Akker-Scheek I, Diercks RL. No difference in effectiveness between focused and radial shockwave therapy for treating patellar tendinopathy: a randomized controlled trial. Knee Surg Sports Traumatol Arthrosc. 2014;22(9):2026-32.
18. Vetrano M, Castorina A, Vulpiani MC, Baldini R, Pavan A, Ferretti A. Platelet-rich plasma versus focused shock waves in the treatment of jumper's knee in athletes. Am J Sports Med. 2013;41(4):795-803.
19. Visnes H, Hoksrud A, Cook J, Bahr R. No effect of eccentric training on jumper's knee in volleyball players during the competitive season: a randomized clinical trial. Clin J Sport Med. 2005;15(4):227-34.
20. Wang CJ, Ko JY, Chan YS, Weng LH, Hsu SL. Extracorporeal shockwave for chronic patellar tendinopathy. Am J Sports Med. 2007;35(6):972-8.
21. Warden SJ, Metcalf BR, Kiss ZS, Cook JL, Purdam CR, Bennell KL, Crossley KM. Low-intensity pulsed ultrasound for chronic patellar tendinopathy: a randomized, double-blind, placebo-controlled trial. Rheumatology (Oxford). 2008 Apr;47(4):467-71.
22. Willberg L, Sunding K, Forssblad M, Fahlström M, Alfredson H. Sclerosing polidocanol injections or arthroscopic shaving to treat patellar tendinopathy/jumper's knee? A randomised controlled study. Br J Sports Med. 2011;45(5):411-5.
23. Young MA, Cook JL, Purdam CR, Kiss ZS, Alfredson H. Eccentric decline squat protocol offers superior results at 12 months compared with traditional eccentric protocol for patellar tendinopathy in volleyball players. Br J Sports Med. 2005;39(2):102-5.
24. Zwerver J, Hartgens F, Verhagen E, van der Worp H, van den Akker-Scheek I, Diercks RL. No effect of extracorporeal shockwave therapy on patellar tendinopathy in jumping athletes during the competitive season: a randomized clinical trial. Am J Sports Med. 2011;39(6):1191-9.
